# Supporting retention in HIV care through a holistic, patient-centred approach: a qualitative evaluation

**DOI:** 10.1186/s40359-022-00722-x

**Published:** 2022-01-29

**Authors:** Cathrine Chinyandura, Anele Jiyane, Xolani Tsalong, Helen E. Struthers, James A. McIntyre, Kate Rees

**Affiliations:** 1 Anova Health Institute, Johannesburg, South Africa; 2 Department of Sociology, University of the Witwatersrand, Johannesburg, South Africa; 3 Division of Infectious Diseases and HIV Medicine, Department of Medicine, University of Cape Town, Cape Town, South Africa; 4 School of Public Health and Family Medicine, University of Cape Town, Cape Town, South Africa; 5 Department of Community Health, School of Public Health, University of the Witwatersrand, Johannesburg, South Africa

**Keywords:** Case management, Retention, Treatment adherence, Holistic, Patient-centered care

## Abstract

**Background:**

HIV is a complex disease which affects different facets (social, economic, physical, emotional and spiral) of an individual’s life, making the goals of retention in care and adherence to treatment difficult to achieve. Holistic patient-centred approaches to providing care for people living with HIV bind together economic, social, emotional and physiological aspects and have the potential to improve retention in care and ART adherence. Case management is a holistic, patient-centred approach which is increasingly being implemented in the management of chronic illnesses.

**Methods:**

We conducted a qualitative study based on semi-structured interviews with key informants (retention officers and social auxiliary workers) and patients. A total of 60 patients and 17 KIs (11 retention officers and 6 social auxiliary workers) participated in the study. The study was conducted in Johannesburg District, Gauteng province, South Africa. Key informants (KIs) and patients were drawn from 8 health facilities located in four management clusters of the district.

**Results:**

The findings identified facilitators and barriers to adherence and retention in care, and demonstrated that case management offered holistic, patient-centred services which patients considered to be beneficial to their well-being and helped them overcome some of these barriers. The success of case management was driven by its holistic and patient-centred approach, which extended the focus to patients’ non-clinical needs which impact on their quality of life. Complex interacting barriers and facilitators at different levels influenced implementation of the model and its outcomes.

**Conclusion:**

Holistic approaches such as case management have a strong potential to improve retention in care and adherence to ART. HIV is a complex disease which impacts different facets of an individual’s life, hence requires holistic care to address all facets. Health systems need to transition towards holistic care to ensure that some patients do not slip through the cracks, improve patient outcomes and efficiency.

## Background

Retention in care and adherence to antiretroviral treatment (ART) are critical elements in achieving the 95:95:95 vision [1]. Ensuring maximum adherence and retention remains a major hurdle in the management of HIV. To address poor ART adherence and attrition, several strategies and interventions are being implemented in different settings across the world. HIV is a complex disease which affects different facets of an individual’s life. It is inextricably connected to an individual’s social, economic, physical, emotional, and spiritual wellbeing [2]. People living with HIV (PLHIV) are often vulnerable to poverty, stigma and discrimination, inequality, depression, feelings of guilt, prejudice and anxiety, making the goals of retention in care and adherence to treatment difficult to achieve [3, 4]. Holistic patient-centred approaches to providing care for people living with HIV bind together economic, social, emotional and physiological aspects and have the potential to improve retention in care and ART adherence, and therefore reduce morbidity and mortality [2].

### Physiological impact

ART has multiple physiological impacts. PLHIV are susceptible to micronutrient deficiencies which in turn increase the risk of opportunistic infections [5]. Study findings indicate that PLHIV have an increased risk of cardiovascular, gastrointestinal and renal complications and metabolic disorders [6].

### Psychological impact

There is evidence that PLHIV experience heightened mental health challenges compared to the general population [7]. Diagnosis of HIV, living with a chronic illness, regular medical appointments, life-time treatment, side effects, physical pain, relationships, and stigma can all be sources of emotional distress. Living with HIV can bring feelings of shame, fear, guilt, anger, worry, grief and sadness. PLHIV often suffer from depression and anxiety [3, 8]. Children and adolescents living with HIV are likely to face an increased burden of mental and behavioural disorders compared to adults [9]. Some medicines for HIV have known side effects which impact on psychological health including insomnia, nightmares and anxiety [10]. If unresolved, and without correct care and support, emotional distress can have devastating effects on retention in care and treatment adherence [8]. Stigmatization can lead to non-disclosure which is associated with poor ART adherence [11].

### Economic impact

HIV threatens the economic well-being of PLHIV and their families due to depletion in income, savings and productive assets, weakened family and societal support systems, increased health care expenditures (hospitalization costs, medicines, travel, medical investigations/tests), increased indebtedness and decreased participation in education [12]. Loss of and interruption of work due to sickness and/or stigmatization result in reduced earnings. Reduced income and savings hinder investment in business and education [4]. These economic burdens hamper retention in care and adherence to treatment, especially considering the opportunity costs of HIV care.

### Holistic and patient-centred approaches

Holistic care is a system of care which promotes cooperation of different stakeholders to achieve optimal physical, psychological, social and spiritual well-being [2]. A holistic approach is premised on the understanding that the whole patient is greater than the sum of the parts [13]. The provision of holistic health care is integral to the philosophy of nursing. Holistic approaches shift the focus from health systems designed around diseases to systems that place people at the centre [2]. Holistic approaches, which are delivered in a person-centred manner, have the potential to improve the quality of life for PLHIV. A holistic approach to care for PLHIV has been found to improve various health and well-being outcomes, including viral suppression and quality of life [14]. The approach requires an interpersonal and interactive relationship with the patient and multidisciplinary collaboration to realise optimal management of the disease [13].

### Case management

Case management is a holistic, patient-centred approach which is increasingly being implemented in the management of chronic illnesses. It promotes individualised health and wellness through addressing clinical, socio-economic and psychosocial needs that affect the functioning and well-being of a patient and his/her family [15]. While case management models vary, their overarching goals and core principles are similar: to provide client-centred services which promote and support autonomy and self-sufficiency, and enhance the functioning status of patients and families. It incorporates proven strategies to strengthen treatment adherence and maximize retention in care.

Research suggests that case management is an effective approach for addressing the complex needs of chronically ill clients [16]. It can help improve quality of life, satisfaction with care and use of community-based services [17, 18]. In HIV management, the model has been found to decrease mortality, unmet needs and risky behaviours and improve health outcomes, treatment adherence, and linkage to and retention in care [19, 20]. There is evidence that case management is positively linked to the uptake of medical and social services, significant increase in patient CD4 counts and successful linkage to HIV care [21, 22].

Anova Health Institute, in partnership with the Department of Health, is implementing a case management model in Johannesburg, South Africa. Anova is the PEPFAR District Support Partner for the City of Johannesburg. The goal of the model is to increase care engagement and treatment adherence of patients. The model utilizes a team of case managers (retention officers) who are trained lay counsellors based at health facilities supported by social auxiliary workers to provide patient-centred supportive services to targeted HIV patients. The model targets high-risk HIV patients: newly diagnosed, youth, children, pregnant women, men, and the disengaged. Patients are recruited through different strategies: health talks, referrals from clinicians (after initiation of ART) and other departments and stakeholders [youth, antenatal care (ANC), community-based organizations (CBOs) and non-governmental organizations (NGOs)]. Patients are enrolled in case management for 6 months following ART initiation or re-initiation, after which they are offered differentiated service delivery mechanisms. Retention officers work with the patient to plan their consultation visits which are held at the health facility. In the consultation meetings, retention officers discuss different aspects including: treatment literacy, side effects, nutrition, disclosure, substance abuse, pregnancy, safe sex, and gender-based violence (GBV). A record of each meeting’s discussions is kept in a patient file. Retention officers also telephonically follow-up patients and send reminders of their consultation meetings. Through regular intensive support, the retention officers work with patients to ensure treatment adherence, facilitate access to needed HIV and non-HIV-related services, navigate the health care system and to function independently. The model interventions are customized to a patient’s needs and, by building rapport with a patient, retention officers are able to gain insights into and assist the patient on psychological, social, economic and spiritual issues which influence their health. We conducted a qualitative evaluation aiming to understand patients’ and providers’ perceptions and experiences of the intervention, including facilitators and barriers to implementation.

## Methods

### Study setting

The study was conducted in Johannesburg District, Gauteng province, South Africa from October 2019 to June 2020. Case Management was introduced in March 2019 and during the study period, 25 health facilities were implementing the model. Key informants (KIs) and patients were drawn from eight health facilities located in four management clusters of the district. A sample of eight facilities was chosen based on best and worst performing facilities with regards to retention in care. By December 2019, the selected eight facilities had a total of 3 521 patients on ART. The retention officers and social auxiliary workers are employed by Anova Health Institute, a South African non-profit organization (NPO) which is a key implementing partner to the Department of Health (DoH) providing technical assistance and direct service support to the HIV/AIDS and TB programme as a PEPFAR District support partner, through USAID.

### Study design and participants

We conducted a qualitative study based on semi-structured interviews with KIs (retention officers and social auxiliary workers) and patients. Convenience sampling was used to select patients while purposive sampling was used to select KIs. Retention officers assisted in the recruitment of patients to participant in the study. Patients were selected based on their availability and willingness to participate in the study. Retention officers and social auxiliary workers working in the selected health facilities were all sampled for the study on the basis of their experience and expertise. A total of sixty patients and seventeen KIs (11 retention officers and 6 social auxiliary workers) participated in the study. Interviews were conducted by research staff, that had not been involved in programme implementation. Interviews were conducted in English and local languages (Zulu, Xhosa, Sepedi, Tsonga and Venda) and audiotaped for subsequent transcription, with simultaneous translation into English. Semi-structured interviews were used to collect data from both KIs and patients. The semi-structured interviews provided rich information as they allowed participants the freedom to freely share their experiences, perceptions, views and insights in greater depth. The method also allowed the researchers flexibility, probing for clarity and deeper understanding. The KI semi-structured guide covered the following topics: recruitment process, services, coordination of care, communication, effectiveness and lessons learned. The patient guide covered the following topics: demographics, client (services, life changes, required services and support, service improvement) and treatment experiences (HIV treatment journey, perceptions on treatment, barriers to treatment, treatment support). Interviews with patients lasted between 30–45 min while with KIs the duration was between 45–60 min. Data collection was conducted between October and December 2019.

### Ethical approval

All methods were carried out in accordance with relevant guidelines and regulations. Written informed consent was obtained from all participants before conducting the interviews. Ethical approval was obtained from the Human Sciences Research Council (HSRC) REC 3/22/08/18 and the Johannesburg District Research Committee.

### Data analysis

The interviews were transcribed and imported into NVivo 12 qualitative data analysis software. The software was used for coding, categorization and identification of themes. Thematic analysis was used to analyse the qualitative data. Two research facilitators transcribed the data, and the generated transcripts were independently reviewed line by line before coding. Coding was done in a two-step process, with one team involved in the inductive generation of initial codes, categories and themes and the second team independently reviewing the codes, categories and themes. The first phase involved open coding; the first team of coders independently examined the textual data line by line and assigned codes to the data excerpts. In this phase the assigned codes were tentative and subject to change as coding progressed. The second phase was categorization, where the codes were grouped into sub-categories and the sub-categories into higher categories. Each sub-category was composed of codes that were similar, in terms of topic or general concept. Categories and codes were re-examined resulting in some codes and categories being re-named, re-coded, merged and re-categorized. The reanalysis of the data enabled finding emerging patterns and themes. To increase reliability, the multiple team members reviewed the coding together in order to have consensus on the codes, categories and themes. Interview guides and field notes were also used to facilitate reflexivity and to ensure the consistency of the processes of coding. The data set was then reviewed by two independent team members. The emerging themes were further refined and merged by both teams to create final themes. The final codes and categories were used to construct the final narrative.

## Results

Our findings identified facilitators and barriers to adherence and retention in care, and demonstrate that participants valued the patient-centred services they received through case management, which allowed them to overcome some of these barriers. In addition, the study identified facilitators and barriers to holistic patient-centred care. KIs highlighted that case management addressed issues which were often neglected by clinicians.

## Case management holistic and patient-centred services

### Socio-economic support

Economic factors such as poverty and unemployment, lack of food and transport were reported by both patients and KIs as major barriers to retention in care and treatment adherence. Case management was commended by both patients and KIs for directly and indirectly addressing some of these economic challenges. Through case management, patients were able to access food parcels, soup kitchens in their communities and employment opportunities.

#### Food and employment

Lack of food was cited by both patients and KIs as a barrier to treatment adherence. The lack of food was attributed to unemployment and job instability. Through case management, patients were counselled on dietary needs for their condition, assisted to receive food parcels from social workers and linked to community soup kitchens and employment services.

She supports me in terms of what I need to eat so that the treatment will work well. **(Participant 2, male)**

Key informants reiterated that a significant number of the patients were unemployed and were struggling to meet their basic needs such as food and transport. KIs explained that they made efforts to link such patients to various supportive programs at the health facility and in the community.

Most of the clients are not working so they do not have the means to get food, so I refer them to the social worker for assistance. **(Key informant 7)**

#### Family counselling and support

Family stigma and discrimination, homelessness, non-disclosure of HIV status and lack of family support were identified by both patients and KIs as contributing factors to non-treatment adherence. To address these challenges, retention officers provided supportive counselling to patients and families. Extreme cases were referred to social auxiliary workers for further counselling. Homeless patients were also linked to safety shelters.

At first, I was not able to come clean to my husband about my status, she helped me to disclose to him and my husband was so understanding. I have been asking my husband to come to the clinic to test. He has been reluctant to come to the clinic but the case manager told me that she can assist me and I’m happy that the case manager convinced him to come. **(Participant 9, female)**

Some of my patients ended up disclosing even though it’s not with the whole family, but they have identified someone in the family that can support them when we are not around. **(Key informant 5)**

### Physical support

Regimen characteristics such as side effects, pill burden and the complexity of treatment were found to significantly affect treatment adherence. Some patients reported treatment fatigue, while others expressed that they were reluctant to continue treatment due to side effects. Case management assisted patients to deal with such issues by providing treatment literacy and support which helped patients understand benefits of treatment, side effects and how to manage them. In some cases, retention officers assisted patients in accessing required further medical support from clinicians.

#### Management of side effects

Most patients expressed that during their consultation sessions, retention officers had educated them on treatment side effects and how to manage them. When patients experienced severe side effects, retention officers referred them to clinicians for further support.

The first time I took the treatment I was very tired and dizzy. I told my case manager she helped me a lot and referred me to the nurse. **(Participant 6, female)**

### Psychological support

The intervention offered intensive counselling to patients who were in distressful situations, including patients experiencing GBV and emotional distress from any other circumstances. Severe cases were referred to social auxiliary workers and other services for further support.

#### Partner notification and contact testing

The rapport created with patients through supportive counselling and consultations provided a key opportunity for partner notification and contact testing. Patients expressed that retention officers assisted them to bring their sexual partners and/or family members into HIV care.

The service has helped me a lot because we came together with my girlfriend to test and she helped us so now we are taking the treatment together. **(Participant 11, male)**

#### Support for GBV survivors

KIs highlighted that they supported patients who were experiencing GBV to access all the needed services which would assist in navigating their conditions and situations.

There was this one lady who I had recruited for case management and emotionally she was struggling because of the abusive relationship she was in, now she is out of the relationship which caused her to be once admitted at Leratong hospital for attempted suicide. Throughout that whole process I was there to support her. I even referred her to the mental health sister who referred her to a psychologist. So I can say it was beneficial because for me I wasn’t only providing point of care but I was able to refer the patient for further services. **(Key informant 4)**

#### Support for substance use

Findings indicated that some patients experienced emotional distress due to the HIV diagnosis and other HIV-related issues which triggered or worsened substance abuse. Consequently, this resulted in non-adherence to treatment and engaging in risky behaviours. Through case management, patients received counselling and education on the links between HIV and substance abuse. In addition, they were linked to other health and social services for further support. Both patients and KIs expressed that the intervention had assisted in dealing with substance abuse.

Ever since I started attending the case management I feel a lot better even my behavior has changed, I used to drink a lot especially after finding out about my status. Then the case manager has improved my life because she is a person who advises me a lot. **(Participant 5, male)**

Most of my patients’ health issues have improved, for example, I have many who have reduced substance abuse which used to affect their treatment. **(Key informant 3)**

### Facilitators to holistic patient-centred care

Patients appreciated the patient-centred care they were receiving from the case management team. Through the services, they felt humanized, respected and worthy. The active engagement in their health gave them a better understanding of the disease and treatment and motivated them to adopt healthy behaviours to safeguard their health.

#### Responsiveness

Patients indicated that case management was responsive to their needs and preferences. It was an answer to their need for quick visits, private and confidential services. Patients appreciated the intervention for improving their experience of HIV care at health facilities.

I am very satisfied. I can give him 10/10 for everything, and this programme and the case manager give me privacy, the hospital is easily accessible, and he is competent for this work. **(Participant 1, female)**

#### Patient empowerment

Patients commended the retention officers for educating and supporting them to make decisions and participate in their own care. They added that through the education and continuous support they received, they felt empowered and confident to take care of their own health.

I am very thankful to my case manager; she has given me a lot of knowledge on this disease. I feel I can take care of myself and anyone else with HIV. She educated me on a lot of things, some things I never knew. **(Participant 14, female)**

#### Positive staff attitudes

Patients valued the good reception and effective communication they received from the retention officers. Patients reported that they were treated with respect, the retention officers were empathetic and passionate about their work. This made them feel worthy, engaged, acknowledged and want to cooperate with the service providers.

She is friendly, when she talks she is not harsh, she listens, she is patient and I am free to ask about anything. **(Participant 4, female)**

Similarly, KIs expressed that they were noticing positive changes in the patients during their assessments. This included the determination to control their well-being, acceptance of their HIV status, disclosure, physical strength and lifestyle changes.

It has created an opportunity for them to look at life from a different angle or maybe to approach things in a different approach. **(Key informant 5)**

### Health-system related barriers to holistic patient-centred care

Negative staff attitudes, poor communication, long waiting times to get service, lack of privacy and confidentiality, and poor coordination of care were identified as health-system related barriers to holistic patient-centred care.

### Negative staff attitudes

Findings showed that negative staff attitudes which involved communicating with patients in a rude, belittling and disrespectful manner hindered patient-centred care. Patients’ concerns were confirmed by KIs who expressed that the negative staff attitudes were impacting negatively on retention. Patients applauded retention officers for being sensitive, warm and polite.

It is the attitude from the staff in the clinic that makes them to default. For instance, one client told me he was mistreated the last time he came to the clinic. He said that the lady that was helping him shouted at him in front of other patients. He defaulted for 8 months because of staff attitudes. **(Key informant 7)**

### Long waiting times

Long waiting times were found to result in dissatisfaction and decreased willingness to seek services. The long waiting times made patients question the competency of staff.

One thing about here, when you come to the clinic we wait for a long time. Sometimes in the filing line, I don’t know how they work, sometimes you come first but they will call someone who came after you. Sometimes you come here as early as 7 a.m but it still doesn’t make any difference. **(Participant 5, male)**

### Lack of privacy and confidentiality

Lack of privacy was cited as a barrier to patient-centred care. Patients disliked waiting for services in open waiting rooms where they were seen by everyone. Consequently, patients preferred getting services at health facilities located a distance from their immediate communities to avoid meeting people they knew. However, this sometimes resulted in incurring high transport costs. Patients were also concerned that service providers sometimes directly or indirectly revealed confidential information in front of other patients. This resulted in patients losing trust in service providers and unwillingness to access the much-needed health services.

I would like to be transferred to another clinic because I stay here and people when they see me sitting outside the benches waiting for her, they will know about my status and I do not like that. **(Participant 3, female)**

There is no confidentiality in this facility, space is a challenge. Everyone else comes in and out so sometimes there is that feeling that our privacy is invaded. **(Participant 17, male)**

## Discussion

The findings indicate that holistic, patient-centred services such as case management have the potential to improve retention in care and treatment adherence. The success of case management was driven by its holistic and patient-centred approach, which extended the focus to patients’ non-clinical needs which impact on their quality of life. This was achieved despite challenges posed by a health-care system which does not promote holistic care. The model provides a framework which can help facilitate transition of the health care system towards holistic care. The study shows that patients are willing to be active partners in their health if they are empowered. Empowered patients can reduce work burden on service providers which consequently improves efficiency. The need for patient empowerment within the context of chronic disease management is well recorded [23–25]. In addition, the study shows that holistic models such as case management are important in identifying and plugging ‘leaks’ through which patients become disengaged from the health care systems. The model is therefore valuable for health systems strengthening.

The patient-centred approach fostered self-empowerment and accountability. The personalized care made patients feel worthy, respected and motivated to take charge of their well-being. Patients reported improvements in physical functioning, quality of life, mental health and self-esteem since joining case management. Observational studies have shown that patients prefer patient-centred care and those who receive it report enhanced health outcomes [26]. Patient centeredness is linked to increased patient engagement, satisfaction and compliance, improved quality of life and reduced patient anxiety [27–29]. By keeping the patient at centre stage, case management ensured that patients actively participated and shared in decision making of their own health. Active involvement of the patients in their treatment and well-being created empowered patients who can manage their illness with confidence.

The need to adopt holistic and patient-centred care is not a new concept but integral to the philosophy of nursing. The WHO definition of health emphasizes holistic patient care and patient autonomy. In South Africa, the HIV/AIDS/STI Strategic Plan for South Africa, 2017–2022 outlines that “holistic, integrated, people-centred care and support” is crucial in reducing HIV-related morbidity and mortality [30].

Although holistic and patient-centred care have been shown to have better patient outcomes, the health care system in South Africa which operates in silos does not promote holistic care. The silo model which creates vertical areas of functional specialization contributes to fragmentation in care and the task-oriented mentality which characterize the model makes it easy to lose patients. The compartmentalisation creates disjointed work processes, inhibits synergies, sharing of knowledge and interactions across programmes [31, 32]. This is contrary to the holistic model of care which promotes cooperation and collaboration, integration, coordination and person- centred care. Chronic conditions like HIV should not be addressed in silos as PLHIV often need to access multiple health and social care services which are typically fragmented and poorly coordinated. The model is an attempt to break these silos and transition towards implementation of holistic health services.

The clinical focus of the health system seldom addresses non-clinical facets of a patient’s life which influence their health. The clinical bias results in resources being primarily allocated for clinical interventions and comparatively fewer resources being assigned to non-biomedical interventions [33]. The clinical orientation is reductionist as it fails to deal with the patient in a holistic manner. While clinical interventions are crucial in the management of HIV, their implementation and effectiveness is often dependent on socio-economic factors, hence the need to ensure that they do not operate in isolation [14]. There is need to move away from being clinical-centric to a more holistic patient model which provides care to the ‘whole’ patient. Transitioning towards holistic, person-centred care is critical to achieve efficiency and effectiveness and better patient outcomes.

Moving forward, it is recommended that, an improved monitoring and evaluation framework is implemented integrating case management indicators in patient management systems. All key stakeholders in case management, particularly at facility level should be trained on the objectives of the intervention, roles and responsibilities of the different stakeholders, relevant policy frameworks, standard operating procedures (SOPs) and guidelines, and performance indicators. Selection of patients for case management should be reassessed to be more nuanced. Evaluation of the intervention should assess clinical and non-clinical outcomes. Future need should consider assessing the impact of case management on clinical outcomes such as virological suppression and lost to follow-up (LTFU). Non-clinical outcomes to be considered include quality of life, economic well-being, labor productivity and mental health.

### Limitations

The study relied on subjective experiences and views of participants meaning the findings cannot be generalized to wider populations and settings. However, the findings offer valuable insights for strengthening of HIV programmes. The study relied on self-reported data which cannot be independently verified and susceptible to selective memory and social desirability biases. Given the variety of responses, we believe the effect of these biases on data accuracy to be minimal. During the study period, outcomes data was not available as the project was still in its early stages. The study did not include all regions of Johannesburg, although we think the selected regions were representative of the district. The study gathered data from Anova Health Institute and beneficiaries of the intervention only and lacks the perceptions and opinions of other stakeholders involved directly or indirectly in case management. It is important that further studies focus on evaluation of clinical and non-clinical outcomes.

## Conclusion

Holistic approaches such as case management have a strong potential to improve retention in care and adherence to ART. HIV is a complex disease which impacts different facets of an individual’s life, hence requires holistic care to address all facets. Health systems need to transition towards holistic care to ensure that some patients do not slip through the cracks, improve patient outcomes and efficiency.

## Data Availability

The datasets used and/or analyzed during the study are available from the corresponding author on reasonable request.

## Abbreviations

AIDS: Acquired immuno-deficiency syndrome
ANC: Antenatal care
ART: Antiretroviral therapy
CDC: Centers for Disease Control and Prevention
DOH: Department of Health
GBV: Gender-based violence
HIV: Human immuno-deficiency virus
HSRC: Human Sciences Research Council
ILO: International Labour Organization
IRB: Institutional Review Board
KIs: Key informants
NGO: Non-Governmental Organization
NPO: Non-Profit Organization
PEPFAR: The United States President’s Emergency Plan for AIDS Relief
PLHIV: People living with HIV
SANAC: South Africa National AIDS Council
SOPs: Standard operating procedures
STI: Sexually transmitted infection
TB: Tuberculosis
USAID: United States Agency for International Development
WHO: World Health Organization
